# Bidirectional genetic selection of behaviors involved in social interaction of Wistar rats

**DOI:** 10.1590/1414-431X2022e11979

**Published:** 2022-04-27

**Authors:** R. Bonuti, S. Morato

**Affiliations:** 1 Laboratório de Comportamento Exploratório, Faculdade de Filosofia, Ciências e Letras de Ribeirão Preto, Universidade de São Paulo, Ribeirão Preto, SP, Brasil

**Keywords:** Rat social behavior, Bidirectional genetic selection, Open field, Anxiety, Exploratory behavior

## Abstract

Bidirectional selection is a procedure in which an arbitrary characteristic is chosen as a selection criterion and animals exhibiting more of this characteristic are bred in one group and animals exhibiting less are bred in another group. The procedure is repeated along generations until the selected characteristic becomes stable, resulting in two strains that are opposite in relation to the chosen characteristic. The present study aimed at selectively breeding rats exhibiting either a high or a low tendency to socialize by using the proximity test. We tested male and female Wistar rats in a square open field with a communicating birdcage, separated by a grid, containing a co-specific rat and coupled on the outside. Subjects that remained more time in front of the birdcage, interacting with the co-specific rat were bred in a group considered of high sociability (SOC+). Likewise, subjects that remained little time in front of the birdcage, with little interaction with the co-specific rat, were bred in a second group considered of low sociability (SOC–). By the 10th generation, the bidirectional selection resulted in SOC+ rats that spent a large amount of time in front of the cage sniffing and rearing in interaction with the co-specific rat and spent less time in the corners, exploring and grooming. It also resulted in SOC– rats that spent a small amount of time in front of the cage sniffing and rearing in interaction with the co-specific rat and spent more time in the corners and used most of their time grooming.

## Introduction

Rats are social animals. They live in well-defined colonies composed by males, females, and pups. Rats tend to be close to other rats even when there are only two of them (1,2). The gregarious and social characteristics of these animals are so important that separation periods can change behaviors to stereotypies or aggression (3-5). In addition, the social interactions between rats include a variety of behaviors ranging from simple interaction (1,6-8), cooperation (9), empathy (10), behavior in colony groups (11), or even interaction between a live rat and a robotic one (12,13). Each of these reports, among others, directs its attention to very different aspects of social interaction between rats. Most studies on rat social behavior have to face one difficulty: rat social behavior is usually studied in pairs of animals, and the behavior of one member always influences the behavior of the other member of the pair. Studying rat interactions in laboratory conditions is a complex task since it is difficult to measure the social behavior of one rat discounting or minimizing the influence of the social behavior of its partner (8,13).

In an attempt to overcome this obstacle, Bonuti and Morato (8) developed a test for social behavior using proximity as a predictor of sociability. The authors used a 120-cm square open field coupled with a small birdcage, with a grid separating the two. The target rat was placed in this modified open-field (MOF) and a partner rat was placed in the cage. The target subject could either explore the MOF or interact with the partner subject through the grid, which prevented full contact between the rats. The main measure was the proportion of time spent interacting with the partner: the more time spent interacting, the more sociable the target rat was. This study found that randomly selected male and female rats exhibited, within a range, different amounts of time interacting with a same-sex partner. One question that could be raised refers to whether these differences in interaction are inherited. One way of approaching this problem is the method of bidirectional genetic selection.

Roughly speaking, bidirectional genetic selection is the selective breeding of animals with a basis on two opposite spectra of the same category: anatomical, physiological, or behavioral (14). As Gomes et al. (15) explains: "Selective breeding is a laboratory technique in which animals are bred in order to modify the frequency of genes underlying a particular phenotype. Mating animals within a population based on the opposite extremes of an observable characteristic will push, over many generations, this particular phenotype in opposite directions, leading to two separately bred lines. This technique has been widely employed to investigate how genes can influence a broad variety of behavioral traits, including defensive reactions associated with emotionality" (page 138).

This technique has been used over the past seven decades. One of the first reports using this technique dealt with mouse sizes (16), obtaining one strain of small mice and another strain of large mice. Since then, laboratory animals have been bred bidirectionally to investigate behavioral characteristics, such as conditioned avoidance (17,18). The technique has also been used to produce strains with high and low characteristics, such as high/low anxiety (14,19-23), high/low freezing (15,24), or even high/low ultrasonic vocalization rates in rat pups (25). All of the above studies used the bidirectional selective breeding to produce two strains exhibiting either an exacerbated or a decreased anatomical, physiological, or behavioral characteristic.

A review of the literature shows that *a*) there are few studies investigating rat social behavior *per se*, and that *b*) bidirectional selective breeding seems to be an adequate procedure to obtain two strains with specific opposite behavior characteristics. Thus, the present study aimed to selectively breed rats exhibiting either a high or a low tendency to socialize by using the proximity test described by Bonuti and Morato (8).

## Material and Methods

### Subjects

Twelve 60-day-old Wistar-derived male rats and 12 60-day-old Wistar-derived female rats were used. The animals came from the Animal House of the Universidade de São Paulo at Ribeirão Preto and were housed in groups of four in polypropylene cages (41×34×17 cm). These 24 animals made up the initial generation (Generation S0). Throughout the experiment, the animals were fed rat chow (Nuvilab, Brazil) and tap water *ad libitum*. The animal room was maintained in a 12-h light/dark photoperiod (lights on at 7:00 a.m.) with the temperature kept between 24 and 27°C. Cage cleaning procedures were performed three times a week and wood shavings were used as bedding. All testing was performed between 7:30 and 11:30 a.m..

### Breeding

After a three-day period of adaptation in the animal room, the starting generation (S0) subjects were submitted to the sociability screening using the test developed by Bonuti and Morato (8) in a MOF. The amount of time interacting with another rat was used as the criterion for selective breeding. The male and female with the highest times of interaction were put to mate in a separate cage, as was the second high-sociability male and female. Likewise, the male and female with the lowest times of interaction and the second lowest were put to mate in separate cages.

This procedure was repeated when the descendants of S0 (S1) were 60 days old and again when the descendants of S1 and of the successive generations reached that age. From generation S3 onward, in order to increase the number of pups, males were put to mate with two females, following the same socialization criterion. Animals bred for high sociability were named SOC+ while animals bred for low sociability were called SOC–. Animals that were not selected for breeding were kept as a reserve, should any problem occur with the selected animals.

After weaning, the animals of one generation were tested in the MOF. Only then were the animals of the previous generation killed with an *ip* barbiturate overdose injection (Thionembutal, 1 g, Abbott, USA).

### Apparatus

The subjects were studied in a MOF (120×120×40 cm) lined with dark brown opaque Formica. It had three conventional walls and a fourth wall with a 20×20 cm opening that contained a bird cage on the outside of the apparatus (34×22×26 cm), where a co-specific rat could be placed. Interaction between the co-specific rat and the focal animal was only possible through the grid. The luminous intensity at the center of the floor was 60 lux. For further details, see Bonuti and Morato (8).

### Procedure

All subjects were tested in the MOF when they were 60 days old. They were tested in pairs of the same sex in 10-min sessions. The target subject was placed in the center of the MOF, while the co-specific rat was placed in the cage. All sessions were recorded by videotape with a camera located 1.75 m above the MOF. The videos were analyzed using X-PloRat, a software developed in our laboratory to record behavior in a computer (26). For this, the image of the apparatus on the monitor was divided into 36 20-cm squares, which allowed for analyzing the frequency and duration, and a place in the apparatus where the behaviors occurred. The 36 squares were grouped in larger areas according to the number of walls surrounding it (for details, see Bonuti and Morato (8)). After each session, the apparatus was cleaned with a 5% ethanol solution and dried with paper towels.

The following behaviors were analyzed: entries into the different squares (later grouped in larger areas), rearing, sniffing, self-grooming, grid gnawing, and time spent interacting with the co-specific rat (our selection criterion for breeding), as measured by the time spent in the square in front of the cage.

All experiments reported here were approved of by the Ethics Committee of the University of São Paulo (Protocol number 15.1.1469.59.9).

### Statistical analysis

All measures from male and female SOC+ and SOC– subjects were compared using the Mann-Whitney rank sum test. In all cases, significance level was set at P<0.05.

## Results

Some problems arose along the five years it took to complete the present work. Some of these were related to breeding while others were related to environmental variables. Three events were related to breeding: some SOC+ animals exhibited a reduced size, some SOC– animals exhibited a high rate of gnawing when tested in the MOF, and some females did not become pregnant.

**Figure 1:**
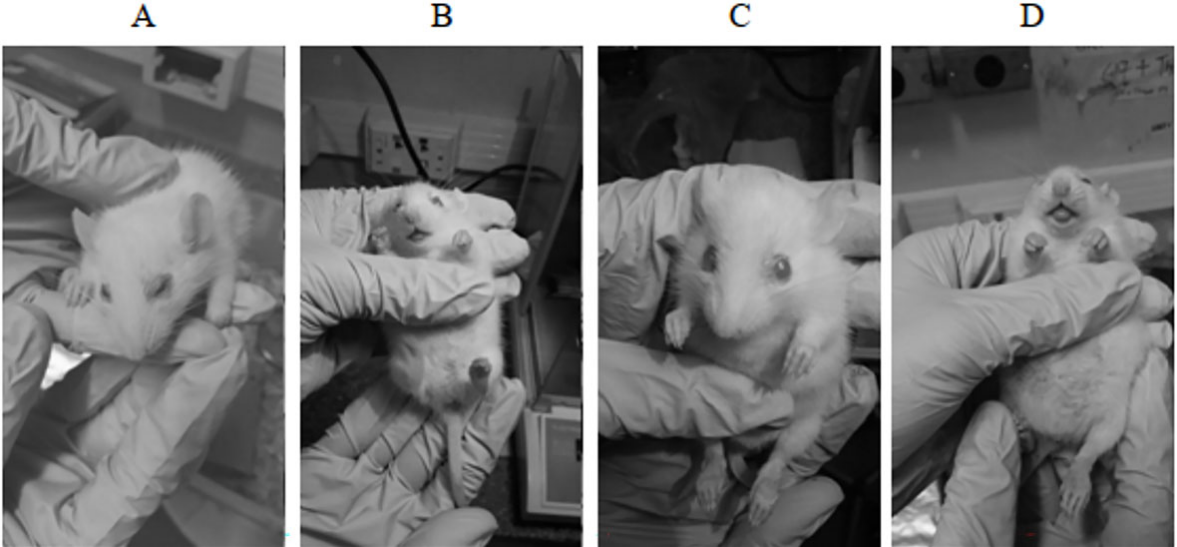
Fifty-three-day-old male with dwarfism from generation S8. Eye inflammation can be seen in **A** and **C** and the lack of teeth in **B** and **D**. The infantile appearance can also be observed.

Breeding events included some SOC+ females (generations S6, S7, and S8) giving birth to pups that, after weaning, exhibited a very small body size compared to the pups in the same litter. At 60 days of age, these pups did not grow like their siblings and maintained the size of a 21-day-old pup. The first ones died right after weaning. When we realized they had no teeth, we fed them powered rat chow and they lived up to two months. Their cranium presented a different shape than their siblings and their tails were shorter. We tried to breed a pair of dwarf rats, but without success. These dwarf rats were not born from generation S9 (Figure 1).

From generation S5 onward, some of the pups exhibited compulsive grid gnawing in front of the cage when tested in the MOF. Since we assessed sociability by time spent in front of the cage, these animals were excluded from the study.

Finally, some SOC– females (in generations S2, S6, S7, and S10) and some SOC+ females (in generations S6 and S9) did not become pregnant and had to be replaced with reserve females of each generation. All substitute females gave birth normally. In spite of these problems, the number of pups born in each generation allowed us to conclude the study. Table 1 shows the number of pups in each generation.

**Table 1:** Pups born from generations S01 to S12 classified as having high sociability (SOC+) and low sociability (SOC–).

Generations	Total	SOC+		SOC−	
		Males	Females	Males	Females
S01	54	18	12	12	12
S02	53	18	18	8	9
S03	52	12	16	14	10
S04	50	11	8	16	15
S05	52	12	13	11	16
S06	54	14	13	14	13
S07	44	13	7	14	10
S08	56	17	15	14	10
S09	53	12	16	15	10
S10	58	24	12	10	12
S11	65	15	12	18	20
S12	62	16	14	13	19

**Figure 2:**
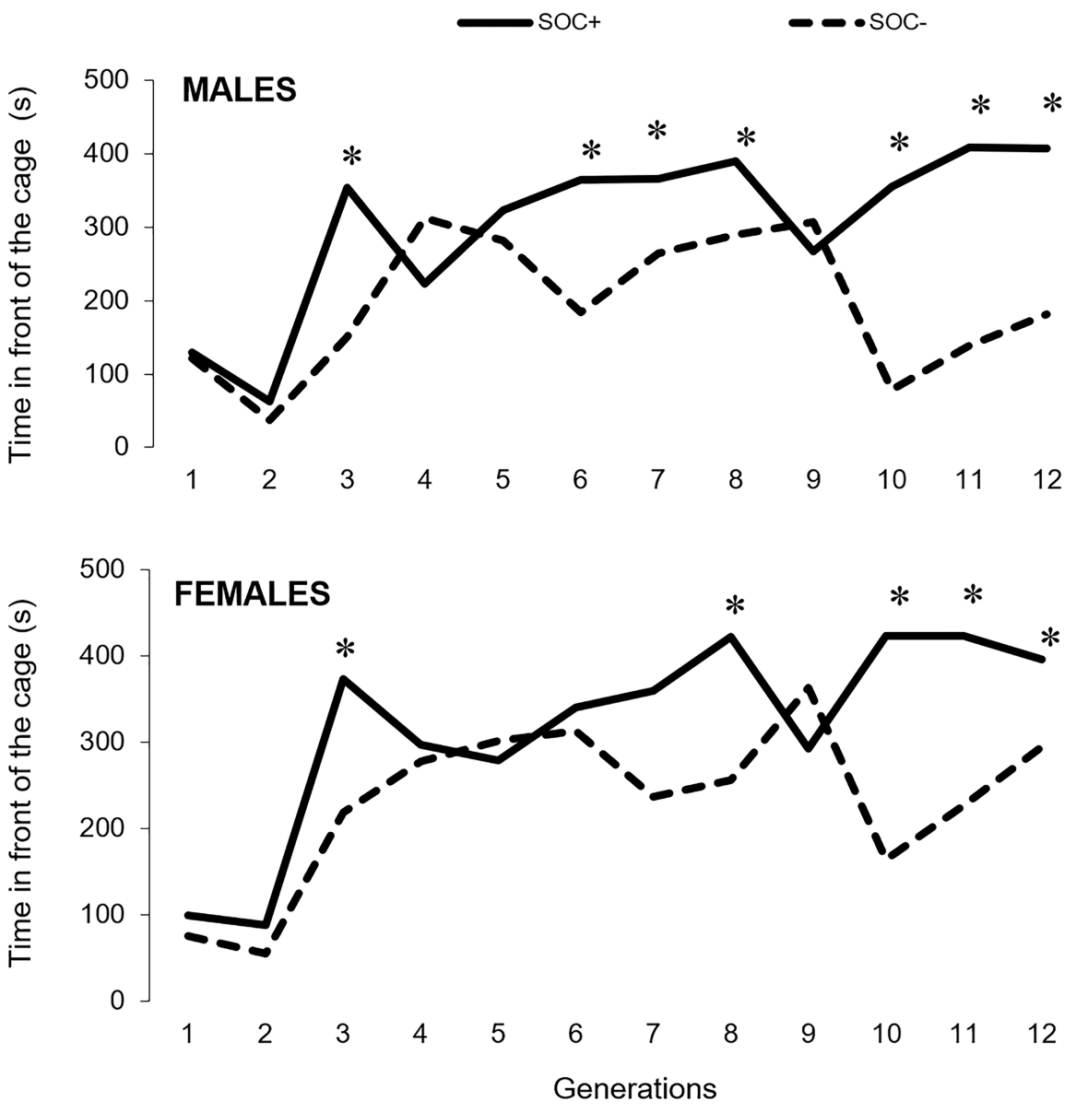
Mean time spent in front of the cage by high sociability (SOC+) and low sociability (SOC–) male (top) and female rats (bottom) in generations S1 to S12. *P<0.05 compared to SOC– (Mann-Whitney test).

Along the five years of the study, three technical problems arose. First, while breeding generation S2, the timer controlling illumination of the animal room malfunctioned and the animals were exposed to continuous light for 15 days. Second, due to repairs in the animal room, pregnant females of generation S3 had to be transferred to another animal room. Finally, the animals of generation S9 were exposed to a large number of rats coming from another vivarium and lodged in the same room and we observed that many animals of this generation lost body weight. It is interesting to notice that these three events coincided with instability in the collected data from the pups from these generations (Figure 2).

Figure 2 shows the time spent in front of the cage by SOC+ and SOC– males and females born in generations S1 to S12. Mann-Whitney test showed a significant generation effect in both males and females (Table 2). SOC+ males spent more time in front of the cage interacting with the co-specific rat than SOC– males in generations S3, S6, S7, S8, S10, S11, and S12. SOC+ females spent more time in front of the cage interacting with the co-specific rat than SOC– females in generations S3, S8, S10, S11, and S12 (Figure 2). In general, it may be concluded that, at the end of twelve generations, the two strains of rats exhibited behaviors with opposite characteristics: long interactions with the co-specific rat in one strain (SOC+) and shorter interactions in the other strain (SOC–).

**Table 2:** Time spent (s) in front of the cage by male and female animals of generations S01 to S12.

Gender	Generations											
	S01	S02	S03	S04	S05	S06	S07	S08	S09	S10	S11	S12
Males	92.00 (0.512)	78.00 (0.760)	34.00 **(0.011)**	119.00 (0.240)	237.00 (0.916)	39.00 **(0.004)**	42.00 **(0.019)**	196.00 **(0.010)**	103.50 (0.745)	269.00 **(0.001)**	174.00 **(0.001)**	199.00 **(0.001)**
Females	64.00 (0.665)	99.50 (0.354)	127.00 **(0.014)**	54.00 (0.723)	170.00 (0.338)	82.00 (0.680)	18.00 (0.107)	17.00 **(0.003)**	83.00 (0.138)	5.00 **(0.001)**	179.00 **(0.001)**	247.00 **(0.002)**

Each cell contains the U value and the P value (within parentheses). Data in bold type indicate a significant difference (P<0.05. Mann-Whitney test).

**Figure 3:**
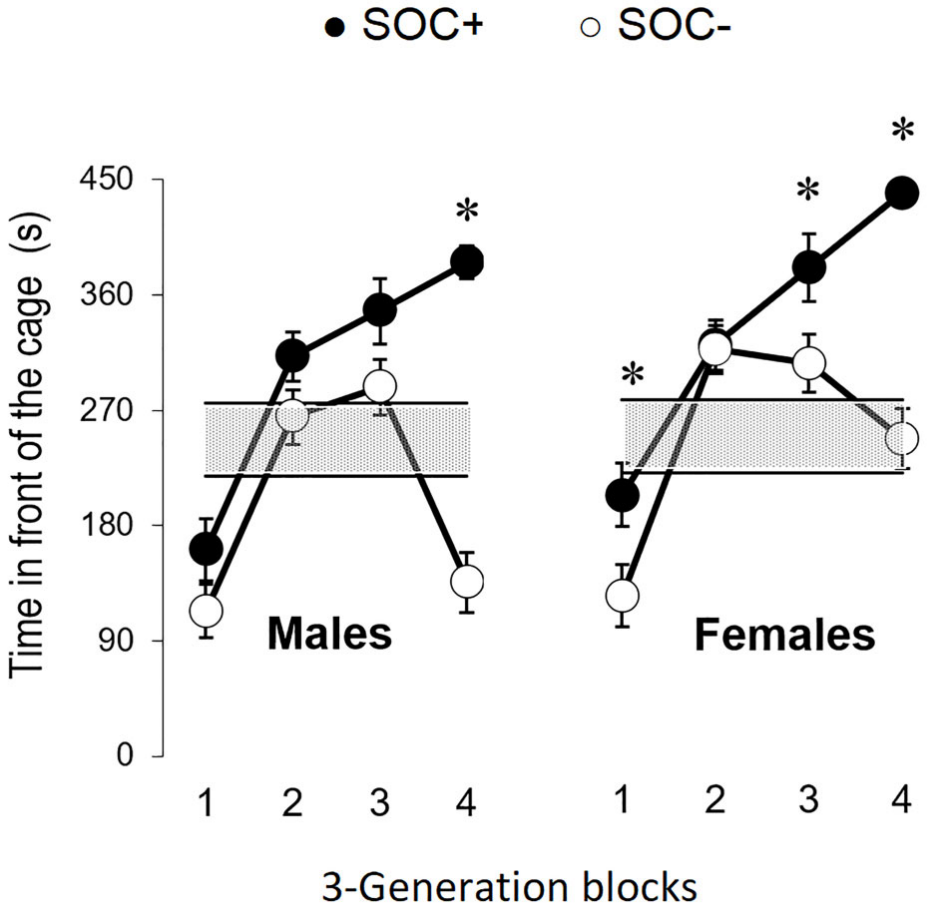
Mean time spent in front of the cage (s) by male (left) and female (right) rats in blocks of 3 generations. Data are reported as means±SE. Gray stripes indicate 1 SE above and below the averages of subjects from generation S00. *P<0.05 compared to SOC– (Mann-Whitney test). SOC+: high sociability; SOC–: low sociability.

Figure 3 shows the time spent in front of the cage in blocks of three generations by SOC+ and SOC– rats. Mann-Whitney test showed an effect of generation in males and females (Table 3). SOC+ subjects in blocks 3 and 4 spent more time in this area than SOC– males and females (Figure 3).

**Table 3:** Other behaviors of males and females in 3-generation blocks.

Behaviors	Blocks							
	Males				Females			
	1	2	3	4	1	2	3	4
Time in front of the cage (s)	840.00 (0.825)	1091.00 (0.227)	624.00 **(0.006)**	1908.00 **(0.001)**	899.50 (0.053)	1020.00 (0.942)	306.00 **(0.018)**	1830.00 **(0.001)**
Body weight (g)	414.00 **(0.001)**	1930.50 **(0.001)**	1565.50 **(0.001)**	8.00 **(0.001)**	779.00 (0.973)	1377.00 **(0.007)**	841.50 **(0.001)**	43.00 **(0.001)**
Time spent in corners (s)	837.00 (0.847)	1409.00 (0.342)	1372.00 **(0.001)**	239.00 **(0.001)**	612.00 (0.297)	929.00 (0.407)	604.00 (0.063)	254.00 **(0.001)**
Time spent close to walls (s)	560.00 **(0.016)**	1555.00 (0.052)	1236.00 **(0.014)**	590.50 **(0.001)**	494.00 **(0.023)**	1187.00 (0.226)	589.00 (0.100)	439.00 **(0.001)**
Time spent in the center (s)	753.50 (0.559)	833.50 **(0.003)**	689.00 **(0.030)**	1575.00 **(0.001)**	737.50 (0.807)	1010.00 (0.852)	492.00 (0.787)	1124.00 (0.408)
Distance ran (m)	718.00 (0.359)	1361.00 (0.533)	1021.00 (0.521)	1005.00 (0.760)	605.50 (0.266)	1187.00 (0.224)	620.00 **(0.037)**	797.50 (0.076)
Time spent sniffing (s)	890.00 (0.489)	341.00 **(0.001)**	222.00 **(0.001)**	1873.00 **(0.001)**	897.00 (0.057)	279.00 **(0.001)**	61.00 **(0.001)**	1937.00 **(0.001)**
Time spent rearing (s)	886.00 (0.513)	832.00 **(0.003)**	763.00 (0.123)	1807.00 **(0.001)**	802.50 (0.355)	594.00 **(0.001)**	398.00 (0.293)	1648.00 **(0.001)**
Time spent grooming (s)	806.00 (0.929)	1721.00 **(0.002)**	1272.00 **(0.006)**	89.00 **(0.001)**	562.00 (0.118)	1166.00 (0.296)	695.00 **(0.002)**	246.00 **(0.001)**

Each cell contains the U value and the P value (within parentheses). Data in bold type indicate a significant difference (P<0.05, Mann-Whitney test).

**Figure 4:**
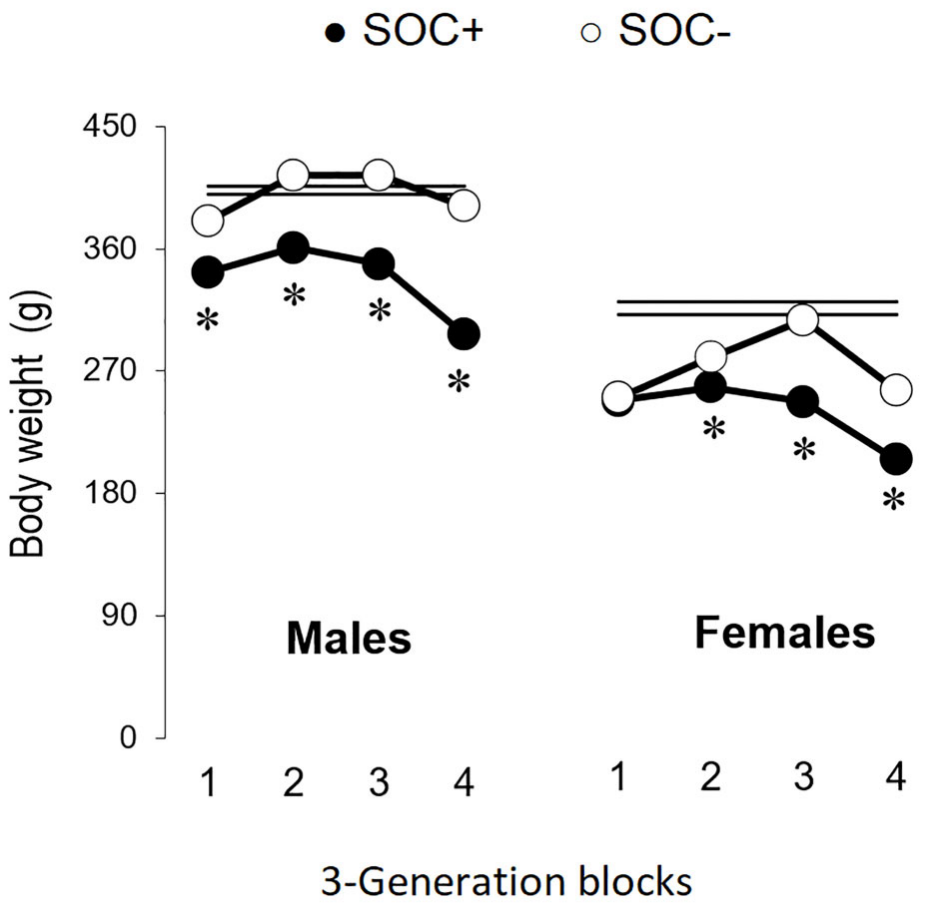
Body weight of high sociability (SOC+) and low sociability (SOC–) male (left) and female (right) rats in blocks of three generations. Data are reported as means±SE. Horizontal parallel lines indicate 1 SE above and below the averages of subjects from generation S00. *P<0.05 compared to SOC– (Mann-Whitney test).

There was an effect of generation on body weight of SOC+ and SOC– males and females in the 3-generation blocks (Table 3). SOC+ males weighed less than their SOC– counterparts in all blocks, while SOC+ females weighed less than their SOC– counterparts in blocks 2, 3, and 4 (Figure 4).

**Figure 5:**
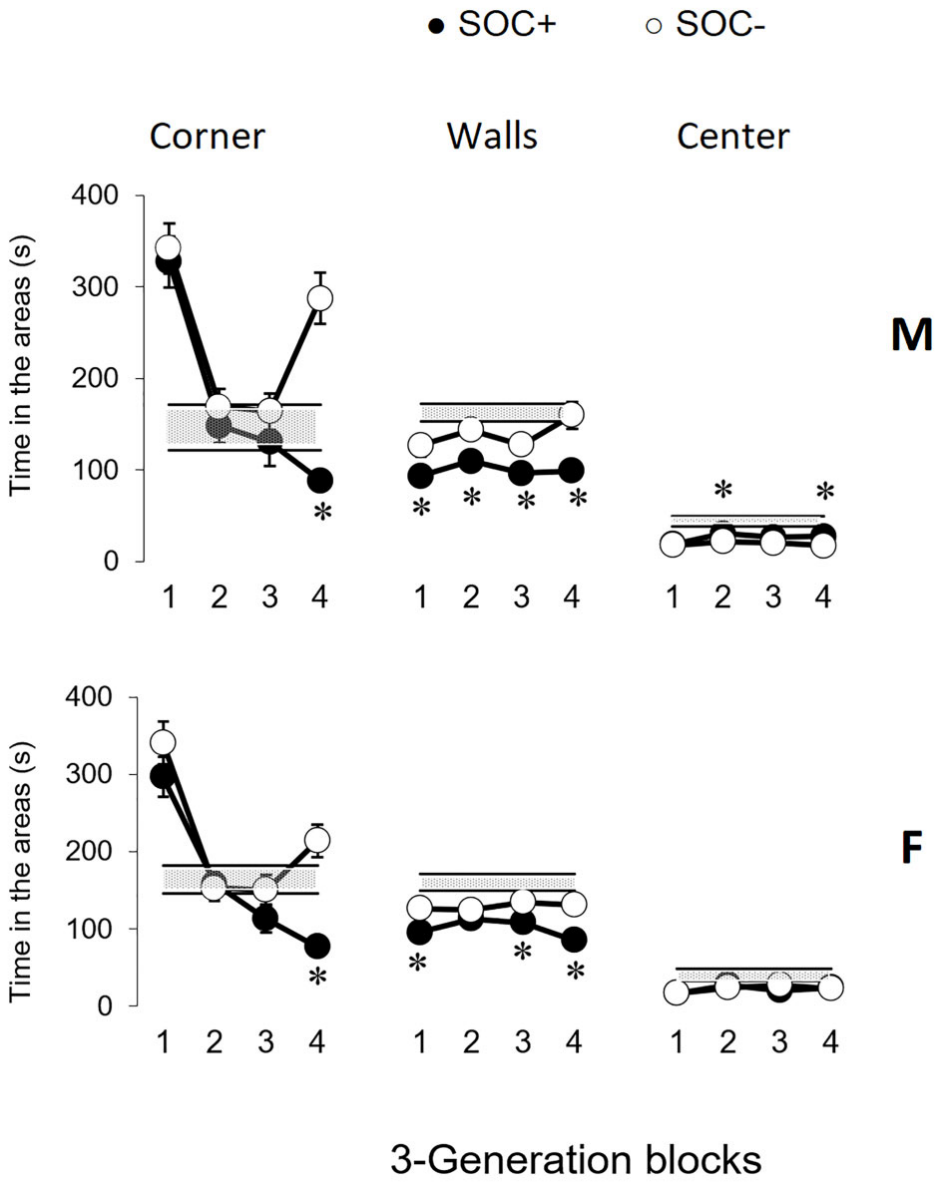
Time spent (s) in corners, close to walls, and in the center by high sociability (SOC+) and low sociability (SOC–) male (M) and female (F) rats in blocks of three generations. Data are reported as means±SE. The gray stripes indicate 1 SE above and below the averages of subjects from generation S00. *P<0.05 compared to SOC– (Mann-Whitney test).

The time spent in the corners, close to the walls, and in the center of the MOF by all the subjects is shown in Figure 5. There was a generation effect both for males and females in time spent in corners and close to walls (Table 3). More specifically, in block 4, both SOC+ males and females spent less time in corners than SOC– subjects. SOC+ males also spent less time in corners and close to walls than SOC– males in block 3. SOC+ subjects spent less time close to walls than SOC– subjects in blocks 1 and 4. Time spent in the center of the MOF had an effect of generation in males, but not in females (Table 3). SOC+ males spent more time in the center than SOC– males in blocks 2, 3, and 4 (Figure 5).

**Figure 6:**
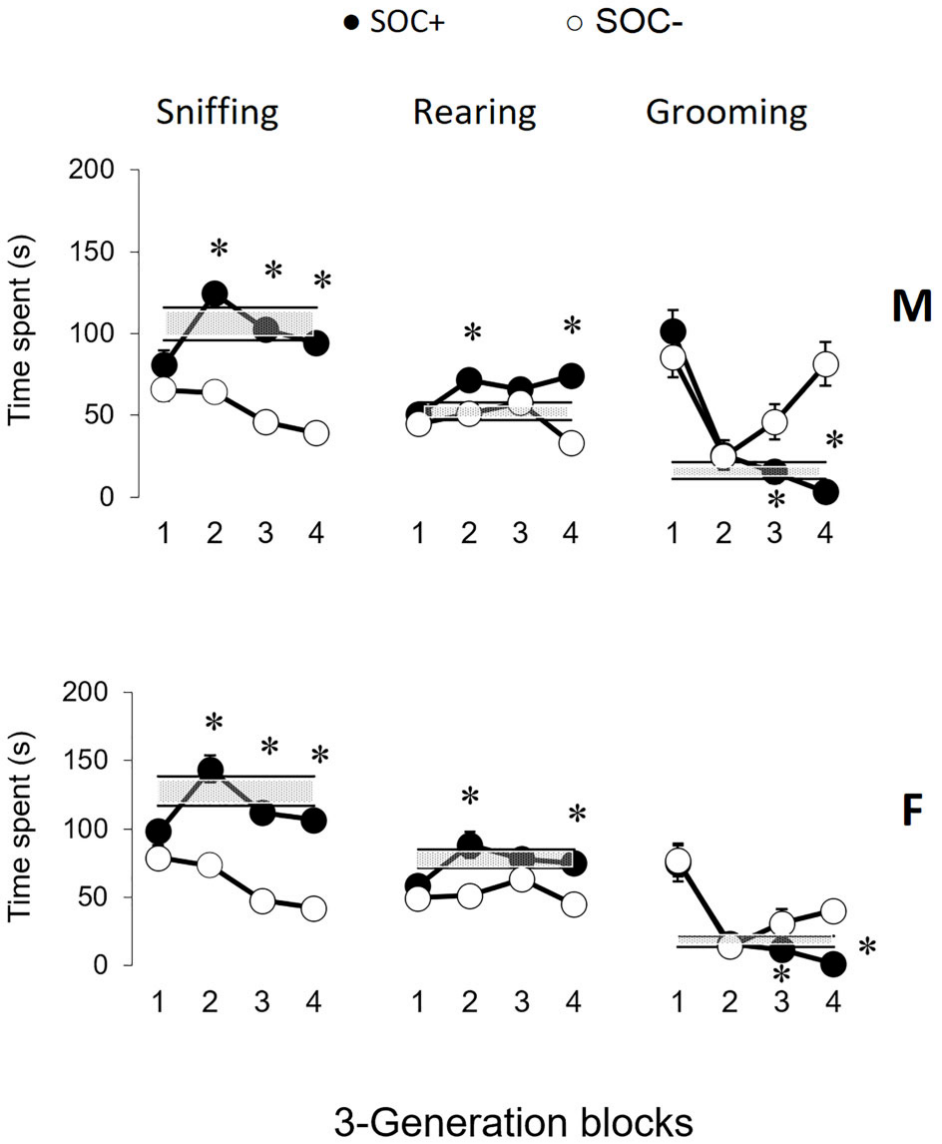
Time spent (s) sniffing, rearing, and grooming by high sociability (SOC+) and low sociability (SOC–) male (M) and female (F) rats in blocks of three generations. Data are reported as means±SE. The gray stripes indicate 1 SE above and below the averages of subjects from generation S00. *P<0.05 compared to SOC– (Mann-Whitney test).

The time spent by the animals sniffing, rearing, and grooming is shown in Figure 6. For all these behaviors, a generation effect was found in males and females (Table 3). SOC+ males and females sniffed longer than SOC– subjects in blocks 2, 3, and 4. SOC+ males and females reared longer than SOC– subjects in blocks 2 and 4. Finally, SOC+ males and females groomed for less time than SOC– subjects in blocks 3 and 4. Also, SOC+ males groomed for less time than SOC– males in block 2 (Figure 6).

## Discussion

### Behaviors directly selected according to the main hypothesis

In general, the data presented here indicated that, in spite of the low number the starting subjects, selective breeding of rats with either high or low sociability (as measured by the proximity test described by Bonuti and Morato (8)) was successful and resulted in two distinct strains of rats. The differentiation of the two strains began to be evident in generation S3 and was stable by generation S10. The fact that the two strains were stable in generation S10 is not uncommon in the literature. While some studies reported that some characteristics are rapidly selected (14,25), others reported that some characteristics can take more time to become stable (16,18).

In spite of being effective, the selection process seems to be better defined in strain SOC+ than in strain SOC–. In fact, comparing the time spent in front of the cage of SOC+ subjects and SOC– subjects with subjects of generation S0, it is possible to see that SOC+ animals differentiated more from subjects of generation S0 than SOC– subjects (Figure 3). In addition, SOC– females differed significantly from SOC+ females and remained in the range of the females from the founding generation. On the other hand, even if SOC– females interacted with the female co-specific rat in the cage in a similar way as generation S0 females, they were different in the other measures. For example, SOC– females exhibited an enhanced anxiety profile and spent more time in corners than females of generation S0, which, in turn spent more time in corners than SOC+ females. Males exhibited similar results. It is important to note that the SOC+ strain was submitted to a selection that resulted in behaviors useful for the rat social environment (mating, grouping, cooperation, protection, etc.), whereas the SOC– strain was selected for behaviors contrary to this nature, making mating, grouping, cooperation, etc. more difficult.

### Behaviors not directly selected according to the main hypothesis

The time in front of cage was not the only behavioral alteration triggered by the bidirectional sociability selection. Time spent close to walls and in the center of the MOF by SOC+ and SOC– rats was also different: SOC– animals spent more time close to walls and less time in the center, a result that can be interpreted as increased fear or anxiety (8, 27-31).

However, in comparison with subjects of generation S0, both strains spent less time in the center and close to walls. Also, both strains ran shorter distances than generation S0. SOC+ and SOC– did not differ in these measures. Such a difference from S0 may occur for different reasons: SOC+ decreased running time because they increased the time spent in front of the cage while SOC– decreased running time because they spent more time in the protected areas of corners. A possible explanation for differences between strains may be because SOC– exhibited higher levels of fear/anxiety and thus preferred to remain near vertical surfaces, such as walls (8,29-31). Obviously, the longer time in front of the cage by SOC+ rats was the result of our selection. The same explanation can be applied to the decrease in the distance ran during the sessions.

Concerning the other behaviors, SOC+ subjects sniffed and reared significantly more than SOC– subjects from block 2 until the end of the experiment. Both sniffing and rearing may be related to the exploration of the environment (the MOF) or to social recognition (8,30,32-34). It is interesting that both these behaviors occurred mostly in front of the cage, while the rat investigated the co-specific rat inside it. Also, the fact that SOC– subjects spent less time sniffing and rearing was probably due to the decreased motivation to interact socially rather than due to a decrease in the motivation to explore the environment.

Finally, SOC– subjects increased the time spent grooming compared to SOC+ subjects. This is a complex behavior, with many possible motivations and explanations. Grooming is usually related to situations of stress and/or conflict caused by novel situations (35-38). In this case, since SOC– subjects tended to groom in corners, both measures were correlated and increased after each generation, and thus indicated increased anxiety. On the other hand, SOC+ subjects decreased time spent grooming and in corners with each generation. Thus, SOC+ subjects increased the time in front of the cage and the time sniffing and rearing, interacting with the co-specific rat, suggesting a high motivation to socialize. On the other hand, SOC– subjects increased the time spent in corners and grooming, suggesting an elevated level of fear/anxiety.

We have no explanations for the appearance of dwarf rats in generations S6 to S8 and for their disappearance in generations S9 onward. The dwarf rats did not survive to the testing age of 60 days. However, two of them were tested for sociability at the age of 50 days and their social behavior was similar to their litter siblings.

In summary, the data indicated successful selection of two strains with opposite characteristics in relation to interacting with a co-specific rat: one with more social behaviors and one with less social behaviors. The selective breeding seemed to be more effective in the selection of SOC+, since these animals differed more from the founder generation in terms of selection criteria, in spite of the small number the initial subjects. On the other hand, SOC– subjects spent more time in corners, probably motivated by fear/anxiety, raising the hypothesis of a possible relationship between fear/anxiety and sociability, a problem that has also been raised by other authors (7,8). However, more behavioral studies in other apparatuses that can better explore the emotional components selected along generations may provide a better understanding of social behavior in rats.
